# Metabolic and Endocrine Alterations in Underweight and Normal-Weight Women with Functional Hypothalamic Amenorrhea

**DOI:** 10.3390/jcm14197082

**Published:** 2025-10-07

**Authors:** Karolina Kowalczyk, Iga Szymańska, Olga Zawistowska, Julia Bieńkowska, Agnieszka Drosdzol-Cop, Paweł Madej

**Affiliations:** 1 Chair and Department of Gynecology, Obstetrics and Oncological Gynecology, Medical University of Silesia, 40-211 Katowice, Poland; 2 Student Scientific Society at the Department of Gynecological Endocrinology, Medical University of Silesia, 40-752 Katowice, Poland; 3 Department of Gynecological Endocrinology, School of Medicine in Katowice, Medical University of Silesia, 40-752 Katowice, Poland

**Keywords:** functional hypothalamic amenorrhea, underweight, metabolic alterations

## Abstract

**Background**: Functional hypothalamic amenorrhea (FHA) is a form of chronic anovulation associated with hypoestrogenism. Weight loss, excessive exercise, stress and long-lasting hypoestrogenism lead to infertility and bone loss. FHA also leads to metabolic changes that increase cardiovascular risk in women who otherwise appear metabolically healthy. **Methods**: This was a case–control study assessing metabolic and endocrine alterations in patients with FHA, stratified by BMI into underweight (BMI < 18.5) and normal-weight (BMI 18.5–24.99) categories. **Results**: Women diagnosed with FHA had significantly higher levels of total (193 ± 41.96 vs. 181 ± 28.23 mg/dL; *p* = 0.037) and LDL cholesterol (67 ± 34.89 vs. 63 ± 24.78 mg/dL; *p* = 0.018) compared with healthy controls. HDL cholesterol levels did not differ between groups; however, normal-weight participants in the study group had higher HDL cholesterol than underweight participants (*p* = 0.007). FHA patients had significantly lower HOMA-IR (*p* = 0.001), lower prolactin (*p* < 0.001), and higher cortisol levels (*p* = 0.036). **Conclusions**: Metabolic and endocrine alterations in FHA patients are modulated both by the condition per se and by BMI. FHA influences total and LDL cholesterol, prolactin, and cortisol levels, while BMI primarily affects HDL cholesterol. Both FHA and BMI have a statistically significant impact on HOMA-IR, but neither influences triglycerides or TSH levels. Our findings indicate that the recovery and prevention of metabolic complications require psychological support and consistent weight management.

## 1. Introduction

In functional hypothalamic amenorrhoea (FHA), classified as hypogonadotropic hypogonadism, the pulsatile secretion of gonadotropin-releasing hormone (GnRH) from the hypothalamus is inhibited. This reduces the secretion of folliculotropic hormone (FSH) and luteinizing hormone (LH) from the pituitary gland. If the hypothalamic-pituitary-ovarian axis is blocked, ovarian function is impaired, leading to low estrogen levels, anovulation, and possibly amenorrhea [1,2].

FHA is the most frequent cause of secondary amenorrhea, accounting for roughly 20–35% of cases in women of reproductive age, and approximately 3% of primary amenorrhea cases. Secondary amenorrhea is defined as the absence of menses for more than three cycles in women with previously regular menstruation, or for more than six months in women with irregular cycles [3]. The diagnosis of FHA is made by excluding other causes (including organic or anatomical factors) [2,3], and should be reversible after addressing the underlying causes [2]. The three primary factors contributing to FHA are stress, eating disorders, and excessive exercise. In many cases, the disorder results from a combination of factors [1,2,3]. It is worth emphasizing that functional menstrual disorders are diagnosed in people with a wide range of body weights and body fat levels, and can occur despite a normal body weight [2,3]. Recent evidence also suggests a genetic predisposition to FHA, which may explain why not all women develop FHA in response to stress, for example [4]. A woman’s metabolism, bone health, cardiovascular function, mental health, and fertility can all be negatively impacted by FHA [1,3,5,6].

To better understand the metabolic changes present in patients with FHA, this case–control study divided participants into underweight and normal-weight groups according to BMI. Through this comparison, we aimed to distinguish the effects of body weight from those of FHA per se on patients’ metabolic and endocrine status.

## 2. Materials and Methods

### 2.1. Methods

This case–control study included patients aged 18–35 who were treated at the Gynecology Endocrinology Department in Katowice between 2018 and 2024. The analysis included 329 women, categorized into groups according to FHA diagnosis and BMI. The Ethics Committee of the Medical University of Silesia approved the use of retrospective data from the patients’ files, no. KNW/NWN/0052/KB/271/24. All patients provided written informed consent, and their confidentiality and anonymity were maintained throughout the study.

### 2.2. Patients

The study group comprised 166 patients diagnosed with FHA, while the control group included 163 patients with normal hormonal status and regular menstrual cycles.

FHA was diagnosed in patients with secondary amenorrhea, defined as the absence of menses for more than three cycles in previously regularly menstruating women, or for more than six months in women with irregular cycles. The progesterone withdrawal test was negative in all patients. Estradiol and LH levels were below the reference range for the early follicular phase (20.5 pg/mL and 2.4 IU/L, respectively). FSH levels were below 10 IU/L. To exclude pregnancy or organic causes of amenorrhea, an external and bimanual gynecological examination, along with gynecological ultrasound, was performed in all patients. Ultrasound examinations were performed using a Voluson E8 Expert (GE Healthcare, Chicago, IL, USA), either transvaginally or transabdominally (the latter for women who were not sexually active).

Exclusion criteria for participation in the study included pregnancy, hypergonadotropic hypogonadism, hyperprolactinemia, clinically relevant thyroid dysfunction, diseases associated with hyperandrogenism, and Cushing’s syndrome.

Both the study and control groups were further stratified by BMI into two subgroups: underweight (BMI < 18.5 kg/m^2^) and normal weight (BMI 18.5–24.99 kg/m^2^).

### 2.3. Measurements

The analysis comprised the results of laboratory tests for various parameters, including total cholesterol, LDL cholesterol, HDL cholesterol, triglycerides, prolactin, TSH, morning (7:00 a.m.) cortisol, fasting insulin, and fasting glucose (used to calculate the HOMA-IR—Homeostatic Model Assessment for Insulin Resistance), as well as patients’ BMI.

Blood samples were taken after overnight fasting. Insulin resistance was assessed indirectly using the HOMA-IR index calculated according to the following formula:

HOMA-IR = fasting serum insulin concentration (µIU/mL) × fasting serum glucose concentration (mmol/L)/22.5.

### 2.4. Biochemical Analysis

Serum concentrations of TSH, prolactin, and cortisol were determined by electrochemiluminescence (ECLIA) using Roche reagents on a Cobas 601 analyzer (Roche Diagnostics GmbH, Mannheim, Germany). Serum glucose, total cholesterol, HDL cholesterol, LDL cholesterol, and triglycerides were determined by colorimetry (AU 640 analyzer) using Beckman Coulter reagents (Brea, CA, USA). Insulin concentrations were determined using the chemiluminescence method (CMIA) with Abbott reagents (Alinity instrument; Chicago, IL, USA).

### 2.5. Statistical Analysis

The database was statistically analyzed using the program Statistica 13.3. A *p*-value < 0.05 was considered statistically significant. Between-group differences were assessed using analysis of variance in a 2 × 2 design, while the effect size was estimated by partial eta-square (ηp^2^) and pairwise comparisons using Tukey’s test for unequal group sizes.

## 3. Results

Analysis revealed that patients in the study group were significantly older than those in the control group. In addition, the study group had a significantly lower BMI compared with the control group; however, both mean values were within the normal range.

Table 1 presents the mean age and BMI of the study and control group along with standard deviations (SD). Table 2 shows these values stratified by subgroup.

In our study, statistically significant differences were observed between the study and control groups in the mean values of total cholesterol, LDL cholesterol, fasting glucose, fasting insulin, HOMA-IR, prolactin, and morning cortisol. Table 3 presents the mean values of lipid parameters, fasting glucose, and insulin, HOMA-IR, prolactin, TSH, and morning cortisol for the study and control groups. Table 4 provides a detailed breakdown by corresponding subgroups. Figures S1 and S2 in the Supplementary Materials include corresponding bar charts of the subgroup data.

### 3.1. Lipid Parameters and Insulin Resistance

**Figure 1:**
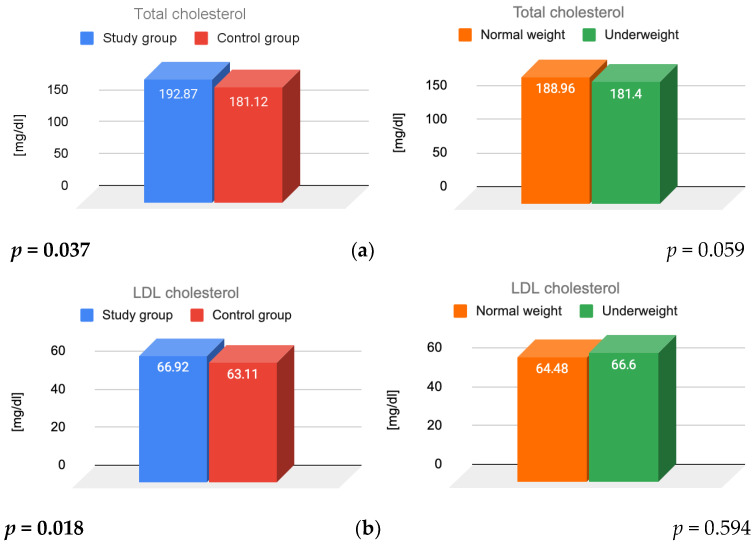

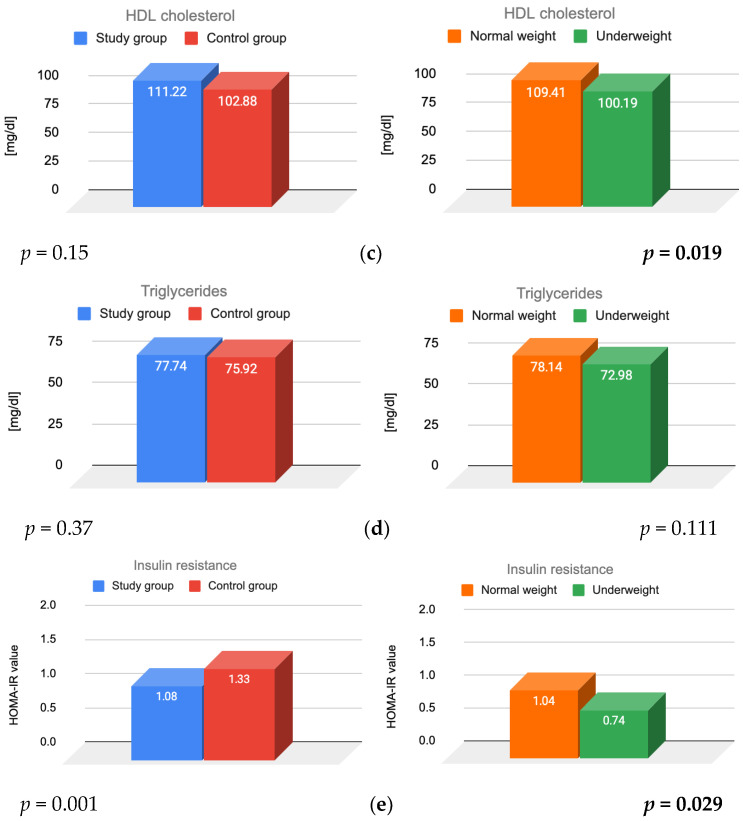
Results for (**a**) total, (**b**) LDL, (**c**) HDL cholesterol; (**d**) triglycerides; and (**e**) insulin resistance, illustrating the main effects of FHA and BMI. LDL—low-density lipoprotein; HDL—high-density lipoprotein; FHA—functional hypothalamic amenorrhea; BMI—body mass index.

The effects of FHA, BMI, and their interaction on lipid parameters and insulin resistance were assessed. Figure 1 presents the results for total cholesterol, LDL cholesterol, HDL cholesterol, triglycerides, and insulin resistance, illustrating the main effects of FHA and BMI.

The analysis showed that women diagnosed with FHA had significantly higher levels of total (*p* = 0.037) and LDL cholesterol (*p* = 0.018) compared to women with regular menstrual cycles. FHA diagnosis was the primary factor influencing total and LDL cholesterol values. No statistically significant differences were observed in mean total (*p* = 0.059) and LDL (*p* = 0.594) cholesterol levels between normal-weight and underweight women. Moreover, the interaction between FHA and BMI was not statistically significant for either total (*p* = 0.17) or LDL (*p* = 0.524) cholesterol.

A significant main effect of BMI was observed for HDL cholesterol levels, with normal-weight women exhibiting significantly higher HDL levels (*p* = 0.019). The primary factor influencing HDL values was BMI. No statistically significant difference in HDL levels was found between the study and control groups (*p* = 0.15). The interaction between FHA and BMI was statistically significant (*p* = 0.033), indicating that BMI moderates the relationship between FHA diagnosis and HDL cholesterol levels. Among women in the study group, those with a normal weight had significantly higher HDL cholesterol levels than those who were underweight (*p* = 0.007). Among normal-weight women, HDL levels were significantly higher in those with FHA compared to those without FHA (*p* = 0.002). When comparing all normal-weight women to underweight women, HDL cholesterol was significantly higher in the first group (109.41 vs. 100.19 mg/dL; *p* = 0.019).

For triglycerides, no statistically significant main effects were observed (*p* = 0.370 for FHA and *p* = 0.111 for BMI), nor was their interaction significant (*p* = 0.794).

Both FHA and BMI had statistically significant main effects on HOMA-IR. Patients diagnosed with FHA had significantly lower HOMA index scores (*p* = 0.001). Normal-weight women exhibited significantly higher insulin resistance scores than underweight women (*p* = 0.029). The correlation between BMI and insulin resistance was statistically insignificant (*p* = 0.190).

### 3.2. Prolactin, TSH and Cortisol

**Figure 2:**
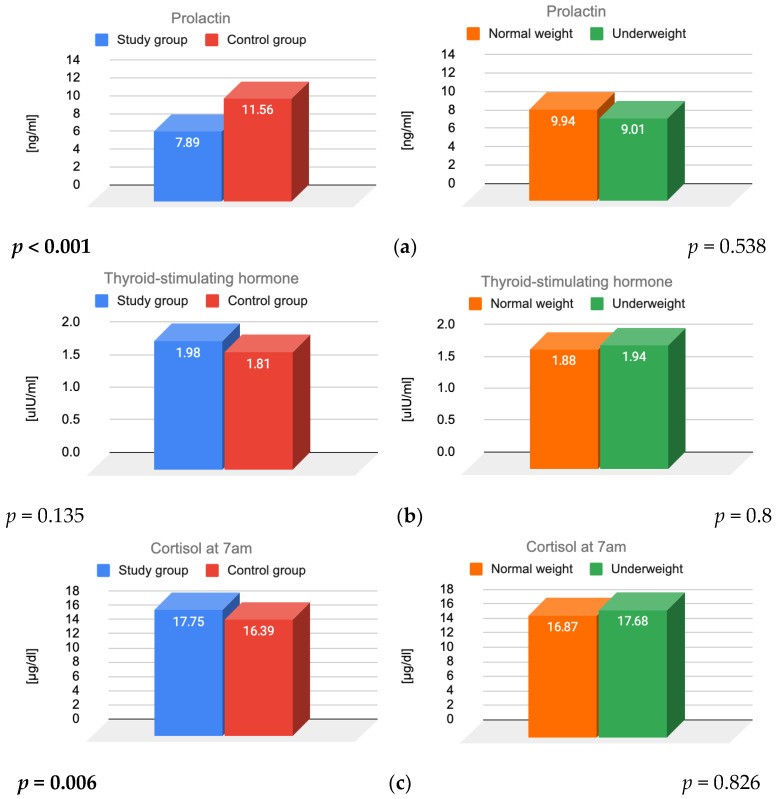
Results of (**a**) prolactin, (**b**) TSH and (**c**) cortisol for main effects of FHA and BMI. TSH—thyroid stimulating hormone; FHA—functional hypothalamic amenorrhea; BMI—body mass index.

The effects of FHA, BMI, and their interaction on prolactin, TSH, and cortisol levels were also assessed (Figure 2).

A significant main effect of FHA was observed for both prolactin (*p* < 0.001) and cortisol (*p* = 0.006), indicating that women diagnosed with FHA had significantly lower prolactin and higher cortisol levels compared to those with regular menstrual cycles. FHA diagnosis was the primary factor influencing prolactin and cortisol levels. Additionally, a statistically significant interaction was identified for prolactin levels (*p* = 0.036).

In contrast, the main effects of BMI on prolactin (*p* = 0.538), TSH (*p* = 0.927), and cortisol (*p* = 0.826) were not statistically significant.

Furthermore, no significant interaction between FHA and BMI was found for TSH (*p* = 0.8) or cortisol (*p* = 0.066).

Post hoc analyses revealed that among women with normal BMI, those with FHA had significantly lower prolactin levels than their healthy counterparts (*p* < 0.001). Such differences were not observed among underweight participants. Within the study group, normal-weight women had lower prolactin levels than underweight women, a pattern not observed in the control group.

## 4. Discussion

### 4.1. Lipid Parameters

Our study found that women with FHA had higher total and LDL cholesterol levels than women without FHA. These results are consistent with a study on young endurance athletes, in which those with amenorrhea exhibited significantly higher total and LDS cholesterol levels compared to those with oligomenorrhea, those with regular cycles, and sedentary controls [7]. Our findings contrast with another study’s results, where total and LDL cholesterol levels in women with FHA did not differ significantly from those in eumenorrheic controls, although absolute levels were slightly higher for some of these biomarkers among women with FHA. In that study, HDL cholesterol and triglyceride levels also showed no significant differences between the study and control group, consistent with our findings [8]. Notably, both of the mentioned studies involved fewer participants, which may make it more difficult to compare results.

In our results, BMI was a differentiating factor for HDL cholesterol levels, which were significantly higher in normal-weight women, while no statistically significant difference was observed between the study and control groups. Moreover, BMI moderated the relationship between FHA diagnosis and HDL cholesterol levels. Women with FHA and a normal weight had significantly higher HDL cholesterol levels than underweight women. For women with FHA, being underweight may exacerbate metabolic issues, leading to a significant decline in the lipid profile. Research focusing specifically on distinctions in lipid concentrations between underweight and normal-weight women with FHA appears to be lacking. Among normal-weight women, HDL levels were significantly higher in those with FHA compared to those without FHA, highlighting the potential combined effect of diet composition and physical activity on the lipid profile in this population. Although other studies have indicated that higher BMI is associated with lower HDL cholesterol levels, these analyses included both women and men, and overweight and obese individuals, thus, groups that differ significantly from ours [9,10].

FHA, characterized by low estrogen, influences lipid metabolism in various ways. Early clinical signs of hypoestrogenemia include menstrual cycle disorders [11]. Due to estrogen’s role in regulating lipoprotein lipase, reduced estradiol increases lipolytic enzymes, shrinking HDL and lowering its effectiveness, even with unchanged or higher HDL levels [12,13,14,15]. This highlights the complexity of the issue, suggesting that research on women with FHA that does not take into account individual classes of HDL cholesterol should be interpreted with caution. Estrogens also increase LDL receptor expression to reduce total and LDL cholesterol levels through the nuclear receptor ERα [14]. Estrogen variations during the menstrual cycle affect PCSK9 levels, which degrade LDL receptors and increase LDL cholesterol [15,16]. The mechanisms described in relation to estrogen deficiency may explain the higher LDL cholesterol levels found in our group of patients with FHA. Interestingly, findings from the SWAN study showed that women with lower BMI may experience the greatest hormone-related increases in LDL cholesterol during menopause [13].

Women with FHA consistently exhibit impaired endothelial function, including reduced flow-dependent dilation (FMD) of the brachial artery, as well as alterations in lipid profiles linked to menstrual dysfunction, which confirms the role of estrogen status [7]. In a follow-up study by the same researchers, amenorrheic athletes showed the lowest FMD at baseline and demonstrated the largest increase after OC treatment, while their lipid profile showed moderate adverse changes consistent with the known effects of second-generation OC [17]. Other data also support this concept. Using the EndoPAT device, which measures endothelial function by assessing NO-mediated changes in vascular tone through fingertip pulse wave amplitude and is independent of age, it was found that young women with FHA exhibited preclinical cardiovascular disease, as indicated by endothelial dysfunction, even when their lipid profiles were relatively normal compared to other groups (eumenorrheic controls and recently menopausal women). This finding indicates that this condition is not solely explained by hypoestrogenemia [8]. Another study suggested that hypothalamic hypoestrogenemia may be a particularly strong risk factor for coronary heart disease in premenopausal women [18].

The results of our study are consistent with existing research indicating that a lack of estrogen can cause metabolic and vascular issues, as our data revealed unfavorable changes in lipid profiles among women with FHA. These findings suggest that FHA may contribute to an increased long-term cardiovascular risk in young women through multiple factors, encompassing both lipid metabolism disorders and endothelial dysfunction.

### 4.2. Insulin

According to our results, women with FHA had considerably lower HOMA-IR values than those without FHA. Normal-weight women had higher HOMA-IR values than underweight women. Both FHA and the BMI had independent effects on insulin resistance. Underweight women with FHA showed the lowest HOMA-IR values, while normal-weight women without an FHA showed the highest.

Our results are consistent with a study in which women with FHA and their age- and weight-matched controls were divided into homogeneous BMI groups—underweight (BMI 15–16, 17–18 (kg/m^2^)) and normal weight (BMI 19–21, 22–24 (kg/m^2^)). In each BMI group, women with FHA had lower insulin levels than those in their respective control groups. Leptin levels were also significantly lower, while cortisol and insulin-like growth factor (IGF) binding protein-1 (IGFBP-1) levels were higher [19].

It is worth mentioning that if we take into account a specific metabolic type of disorder among women with FHA, such as polycystic ovarian morphology (PCOM), these women exhibit higher HOMA-IR values and a significant positive correlation between BMI and insulin resistance [20]. Another study showed that PCOM is associated with reduced insulin sensitivity, regardless of BMI [21]. Among women with FHA, PCOM may occur in more than 44% of those tested [22]. Since selective insulin resistance is a characteristic feature of polycystic ovary syndrome (PCOS), the co-occurrence of FHA and PCOM may weaken the enhanced insulin sensitivity profile observed in FHA, explaining the higher HOMA-IR index in this subgroup [23]. Although PCOM was not specifically assessed in our cohort, these data suggest that metabolic heterogeneity exists among women with FHA.

Another study found that women with FHA had lower glucose and insulin during feeding, with hypoinsulinemia linked to dietary fat, regardless of calorie intake. In this context, insulin secretion appears to be particularly sensitive to the macronutrient composition of the diet. Up to 40% of patients with FHA exhibit bulimic behaviors and restrictive eating habits. When FHA results from restrictive eating, this pattern includes reduced fat intake and increased fibre intake [24]. Because of energy deficiency, energy-saving mechanisms are activated, including a reduction in leptin, glucose, insulin and IGF-1 [25]. Insulin, secreted by pancreatic β-cells, plays a key role not only in metabolic regulation but also in direct hypothalamic signaling [26]. In animal studies, the restoration of normal dietary intake, and the consequent resumption of pulsatile LH secretion, was preceded by an insulin increase [27,28]. Reduced insulin levels in patients with FHA may reflect decreased pancreatic β-cell activity secondary to reduced glucose availability. A combination of various signals may result in the regulation of kisspeptin secretion and subsequent fertility, underscoring the important role of peptides like insulin or leptin [27,29].

The literature provides examples of mechanisms underlying insulin resistance in obese people, helping to explain our results. In obesity, adipocyte hypertrophy activates, inflammatory cytokines and serine kinases, which impair insulin signaling [30,31]. Additionally, insulin’s suppressive effect on lipolysis becomes insufficient, leading to elevated circulating free fatty acids, a condition referred to as adipose tissue insulin resistance. Excess free fatty acids further contribute to insulin resistance in skeletal muscle and liver, partly through the ectopic fat accumulation [32,33]. Adipose tissue is a key source of major adipocytokines, including adiponectin and leptin. Adiponectin, which mediates insulin-sensitizing effects, decreases with increasing adiposity. Conversely, leptin levels increase, which under normal conditions inhibits appetite, stimulates thermogenesis, enhances fatty acid oxidation, decreases glucose levels, and reduces body weight and fat. However, in obesity, leptin resistance develops [34].

In women with FHA, restrictive eating is frequently accompanied by high levels of physical activity. This combination contributes to a body composition with reduced fat mass and relatively higher lean mass percentage, despite lower total body weight, which may explain why underweight women with FHA had the lowest mean HOMA-IR values among all the groups studied [6,35,36,37,38]. Considering it, compared to women without diagnosis, women with FHA may represent a metabolically distinct profile, more insulin-sensitive. Taken together, these findings suggest that insulin sensitivity is closely related to body composition, highlighting the importance of assessing body composition—not just BMI—when evaluating metabolic health. Although we did not directly measure body composition, the lower HOMA-IR values observed in underweight women with FHA support the notion that reduced fat mass and higher lean mass improve insulin sensitivity.

### 4.3. Prolactin

Our study found that prolactin levels were significantly lower in women with FHA. Body mass index did not significantly affect the results in either group, indicating that the findings were unrelated to weight. While elevated prolactin levels are more commonly linked to amenorrhea due to disruptions in ovulation, low prolactin levels can also contribute to menstrual irregularities. In cases of short-term, acute stress, the body may react with increased prolactin secretion as part of an adaptive hormonal response to mobilize body resources. The reduced prolactin levels observed in FHA contrast with the transient hyperprolactinemia seen during acute stress, suggesting that FHA reflects a state of chronic stress adaptation, where prolonged hypothalamic-pituitary axis activation leads to suppressed prolactin secretion.

Studies investigating the effects of different types of stress, acute or chronic, have shown that prolactin secretion is regulated in a manner that depends on both the nature and duration of the stressor. Under acute stress conditions, such as those simulated using the Trier Social Stress Test (TSST), healthy individuals consistently demonstrate a marked but temporary rise in circulating PRL levels [39].

Chronic exposure to sustained stress has been shown to trigger compensatory adaptations within the hypothalamic–pituitary–adrenal axis. These long-term regulatory shifts often result in a blunted PRL response, characterized by diminished circulating PRL concentrations. Evidence from animal studies employing chronic mild stress (CMS) paradigms indicates that persistent activation of stress-responsive pathways may disrupt normal prolactin synthesis and secretion. This suppression of PRL is thought to reflect a broader neuroendocrine recalibration aimed at maintaining homeostasis under conditions of prolonged stress, potentially affecting reproductive, immune, and metabolic functions [40].

The reduced prolactin levels observed across the entire study group may reflect a consequence of chronic adaptation within the hypothalamic–pituitary–adrenal (HPA) axis. Reduced body weight is a chronic stressor for the body and particularly in the context of long-term stress, may lead to prolonged activation of the HPA axis, resulting in adaptive changes within the dopaminergic system—an increase in the activity of the dopaminergic system [40,41].

Prolactin plays various roles in metabolism, immune function, and reproductive health, and its deficiency appears to disrupt these systems. Hypoprolactinemia is correlated with an increased accumulation of visceral fat, which contributes to a higher risk of metabolic syndrome [41].

This condition is associated with higher rates of visceral obesity and dyslipidemia compared to individuals with normal PRL levels. Prolactin plays a regulatory role in pancreatic β-cell function and glucose homeostasis, influencing insulin secretion, β-cell proliferation, and overall glucose metabolism. Low levels of PRL may impair insulin sensitivity, increasing the risk of type 2 diabetes [31,32].

Prolactin is thought to have a direct role in lipid metabolism, as studies have shown an increase in PRL receptor expression during adipocyte differentiation, indicating its potential involvement in the regulation of mature adipocytes [41]. A study on women with hypoprolactinemia exhibited elevated levels of glucose, glycated hemoglobin, triglycerides, uric acid, high-sensitivity C-reactive protein (hsCRP), and fibrinogen.

Interestingly, recent research showed that women with FHA have similar prolactin levels to healthy age-matched individuals. Eating disorders and excessive exercise tend to lower prolactin levels in FHA women, whereas TSH is associated with prolactin levels > 12 µg/L [42]. Prolactin levels in FHA women could be considered as a “sensor” of the hypothalamic–pituitary dysregulation. It seems that prolactin levels in FHA women are mainly influenced by metabolic causes. Our study is consistent with other reports, indicating that hypoprolactinemia in women with FHA may represent a key pathophysiological mechanism underlying adverse metabolic alterations. This highlights the relevance of FHA not only in terms of reproductive function but also for overall metabolic health.

While studies suggest a connection between prolactin and metabolic disorders, prolactin is not typically assessed in metabolic evaluations. Measuring prolactin levels may provide additional insight into endocrine and metabolic risk profiles, particularly in individuals presenting with both reproductive and metabolic abnormalities [41]. Further studies should evaluate the predictive value of PRL for the development of insulin resistance and metabolic syndrome, as well as determine whether normalization of PRL levels is associated with improvements in metabolic parameters.

### 4.4. TSH

In the obtained results, serum TSH levels did not differ significantly between the subgroups. This finding is consistent with previous observations indicating that TSH concentrations in women with FHA typically remain within the normal range or at its lower limit, while levels of free thyroid hormones—FT3 and FT4—tend to be decreased [25,43,44,45]. These changes reflect an adaptive physiological response to stress and energy deficiency—indicating increased negative feedback of thyroid hormones at the hypothalamic level and reduced thyroidal responsivity to TSH.

A likely mechanism is impaired conversion of thyroxine (T4) to the active metabolite triiodothyronine (T3), accompanied by increased production of the inactive form reverse T3 (rT3), which blocks T3 receptors [2,43]. One study revealed that in FHA, a reduced binding affinity of thyroid-binding globulin (TBG) has also been observed, which may explain the discrepancy between normal levels of free T3 and free T4 and decreased levels of total T3, total T4, and thyroid-binding proteins [46].

### 4.5. Cortisol

Our findings showed that cortisol levels were significantly higher in women with FHA compared to the control group. Body mass index did not significantly influence cortisol levels in either group, indicating that the observed effects were independent of weight status. The results are in line with numerous previous studies, which demonstrate that chronic stress leads to increased activity of the HPA axis, consequently resulting in elevated cortisol levels [2,3,4,6,47,48,49,50]. One study used frequent nighttime sampling—it indicated elevated cortisol levels in adolescent and young adult athletes with amenorrhea, compared with eumenorrheic athletes and nonathletes [43]. In the study that compared cortisol levels among amenorrhoeic exercisers, eumenorrhoeic exercisers and nonexercisers, the higher cortisol secretion was associated with lower LH secretion [51].

Under physiological conditions, all hormonal axes are subject to regulation through both positive and negative feedback mechanisms. In each phase of the secretory activity of the hypothalamic–pituitary–ovarian (HPO) and hypothalamic–pituitary–thyroid (HPT) axes, a reduction in hormone concentrations can be observed. Studies show that in the state of FHA the HPA axis is overstimulated at each stage of regulation—increased corticotropin-releasing hormone (CRH) secretion results in an increased secretion of adrenocorticotrophin from the pituitary and cortisol from the adrenal glands [2].

In the absence of stress factors, the secretion of CRH, adrenocorticotropic hormone (ACTH), and cortisol is kept in a circadian, pulsating manner, with peak output in the early morning hours. This physiological secretory pattern is disrupted upon exposure to stressors. During an episode of stress, CRH secretion increases significantly to activate the whole HPA axis. Additionally, other stress mediators are also released to synchronously stimulate the HPA axis [1,5,52].

Although cortisol is commonly used as a biological marker of stress, its interpretation requires caution as it may be dependent on several methodological considerations. The choice of biological matrix has a substantial impact on results: serum and plasma reflect short-term variations, saliva provides a non-invasive measure of free cortisol, while hair cortisol concentrations provide a long-term index of HPA axis activity. Additionally, cortisol secretion follows a circadian rhythm, with peak levels in the early morning and a subsequent decrease during the course of the day [53].

Studies indicate that cognitive-behavioral therapy (CBT) can significantly improve the regulation of the HPA axis, particularly in women presenting with FHA. Evidence was presented in a randomized controlled trial by Michopoulos et al. (2013)—CBT led to a reduction in cortisol concentrations, whereas no such effect was observed in the observation group [44]. Moreover, CBT improved other neuroendocrine and metabolic disturbances associated with FHA [44]. In a recent review, Dobranowska et al. (2024) highlighted CBT as one of the recommended treatment strategies, alongside dietary and lifestyle interventions, aimed at reducing stress and normalizing glucose, triiodothyronine (T3), and cortisol levels, thereby supporting the restoration of menstrual function [54]. Finally, Kyriakidis et al. (2016) emphasized that psychological approaches, including counseling—often incorporating CBT—combined with nutritional interventions appear to improve hormonal status in women with FHA [48].

### 4.6. Strengths and Weaknesses of the Study

The study is distinguished by the comprehensive diagnostic evaluation of the entire cohort of patients within a single tertiary care medical center, ensuring methodological consistency. The whole study group was relatively large, comprising 329 patients, which increases the statistical reliability of the findings. The analysis of participants from the study and control groups according to BMI and the presence of FHA enabled the assessment of the effects of both the disease and body mass on specific parameters, which has not been studied so thoroughly before.

The study lacks an analysis of potential confounding factors, such as physical activity, diet, duration of FHA, substance use, or comorbidities. The analysis did not account for the potential influence of FHA duration on metabolic parameters, and all patients with FHA, regardless of the underlying cause, were treated as a single study group. Conducting the study in a single center may restrict the extent to which the results can be applied to a wider population of women with FHA.

## 5. Conclusions

Metabolic and endocrine alterations in women with FHA are modulated both by the disease per se and by BMI. FHA primarily influences total and LDL cholesterol, prolactin, and cortisol levels, while BMI primarily affects HDL cholesterol. Both FHA and BMI exert statistically significant effects on HOMA-IR, but neither factor influences triglycerides or TSH levels.

Although all metabolic and hormonal parameters examined in the study group remained within normal limits, our findings confirm that these minor metabolic and endocrine disturbances are part of the broader spectrum of this chronic disorder. Functional hypothalamic amenorrhea remains a complex neuroendocrine disorder requiring multidisciplinary treatment. Based on our findings, psychological support and weight management are key for full recovery and the prevention of health complications.

## Figures and Tables

**Table 1:** Mean age and BMI of the study and control groups.

Variable (Mean Value)	Study Group N = 166	Control Group N = 163	*p* Value
**Age** [years]	29.83 ± 6.35	25.65 ± 5.55	**<0.001**
**BMI** [kg/m^2^]	19.74 ± 2.29	20.62 ± 1.9	**<0.001**

**Table 2:** The mean age and BMI values of the subgroups.

Variable (Mean Value)	Study Group		Control Group	
	Normal Weight	Underweight	Normal Weight	Underweight
**Age** [years]	26.12 ± 5.7	24.10 ± 5.08	29.42 ± 5.56	26.88 ± 5.11
**BMI** [kg/m^2^]	21.02 ± 1.64	17.24 ± 0.9	21.17 ± 1.53	17.72 ± 0.65

**Table 3:** The mean values of parameters assessed in the study and control groups.

Parameter	Study Group N = 166	Control Group N = 163	*p* Value
**Total cholesterol** [mg/dL] (<200)	193 ± 41.96	181 ± 28.23	**0.037**
**HDL cholesterol** [mg/dL] (>40)	111 ± 13.99	103 ± 12.44	0.15
**LDL cholesterol** [mg/dL] (<130)	67 ± 34.89	63 ± 24.78	**0.018**
**Triglycerides** [mg/dL] (<150)	78 ± 33.61	76 ± 40.78	0.37
**Fasting glucose** [mg/dL] (74–106)	83.13 ± 8.59	87.78 ± 7.15	**<0.001**
**Fasting insulin** [µIU/mL] (<24)	5.3 ± 3.1	6.11 ± 2.79	**0.014**
**HOMA-IR** [units] (<2.5)	1.08 ± 0.68	1.33 ± 0.64	**0.001**
**Prolactin** [ng/mL] (4.79–23.3)	8 ± 11.1	12 ± 7.62	**<0.001**
**TSH** [µIU/mL] (0.27–4.2)	2 ± 2.11	2 ± 0.86	0.135
**Morning cortisol** [µg/dL] (4.82–19.5)	18 ± 177.81	16 ± 5.21	**0.006**

HDL—high-density lipoprotein; LDL—low-density lipoprotein; HOMA-IR—Homeostatic Model Assessment for Insulin Resistance; TSH—thyroid-stimulating hormone.

**Table 4:** Mean values of assessed parameters by subgroup.

Parameter	Study Group N = 166		Control Group N = 163	
	Normal Weight N = 109	Underweight N = 57	Normal Weight N = 137	Underweight N = 26
**Total cholesterol** [mg/dL]	198.3 ± 44.34	182.49 ± 35.82	181.53 ± 27.94	179 ± 34.29
**HDL cholesterol** [mg/dL]	117.44 ± 35.95	99.32 ± 30.27	103.02 ± 24.82	102.12 ± 24.98
**LDL cholesterol** [mg/dL]	66.2 ± 14.24	68.28 ± 13.52	63.11 ± 12.2	62.92 ± 12.88
**Triglycerides** [mg/dL]	79.5 ± 33.5	74.37 ± 23.44	77.06 ± 27.82	69.92 ± 16.69
**Fasting glucose** [mg/dL]	84.25 ± 7.69	87.70 ± 7.49	80.91 ± 9.82	88.19 ± 5.16
**Fasting insulin** [µIU/mL]	5.68 ± 3.12	6.17 ± 2.78	4.57 ± 2.94	5.78 ± 2.86
**HOMA-IR** [units]	1.04 ± 0.7	0.74 ± 0.6	1.23 ± 0.65	1.14 ± 0.58
**Prolactin** [ng/mL]	7.57 ± 3.75	8.48 ± 4.68	11.83 ± 4.98	10.17 ± 4.69
**TSH** [µIU/mL]	1.97 ± 1	2.01 ± 0.86	1.82 ± 0.81	1.8 ± 0.84
**Morning cortisol** [µg/dL]	17.23 ± 5.35	18.73 ± 6.16	16.57 ± 5.07	15.4 ± 5.24

HDL—high-density lipoprotein; LDL—low-density lipoprotein; HOMA-IR—Homeostatic Model Assessment for Insulin Resistance; TSH—thyroid-stimulating hormone.

## Data Availability

The data presented in this study are available on request from the corresponding author due to ethical reasons.

## Supplementary Materials

The following supporting information can be downloaded at: https://www.mdpi.com/article/10.3390/jcm14197082/s1, Figure S1: Results of total, LDL, HDL cholesterol and triglycerides in the subgroups; Figure S2: Results of insulin resistance, prolactin, TSH and cortisol in the subgroups.

## Abbreviations

The following abbreviations are used in this manuscript:

FHA	Functional Hypothalamic Amenorrhea
BMI	Body Mass Index
LDL	Low-Density Lipoprotein
HDL	High-Density Lipoprotein
HOMA-IR	Homeostatic Model Assessment for Insulin Resistance
PRL	Prolactin
HPA axis	Hypothalamic–Pituitary–Adrenal Axis
TSST	Trier Social Stress Test
CMS	Chronic Mild Stress
TSH	Thyroid-Stimulating Hormone
FT3	Free Triiodothyronine
FT4	Free Thyroxine
T4	Thyroxine
T3	Triiodothyronine
rT3	Reverse Triiodothyronine
TBG	Thyroid Binding Globulin
CRH	Corticotropin-Releasing Hormone
ACTH	Adrenocorticotropic Hormone
HPO	Hypothalamic–Pituitary–Ovarian axis
HPT	Hypothalamic–Pituitary–Thyroid axis
OC	Oral Contraceptives
PCOM	Polycystic Ovarian Morphology
PCSK9	Proprotein Convertase Subtilisin/Kexin Type 9
CMIA	Chemiluminescent Microparticle Immunoassay
hsCRP	High-sensitivity C-Reactive Protein
IGF	Insulin-like Growth Factor
IGFBP-1	Insulin-like Growth Factor Binding Protein 1
ECLIA	Electrochemiluminescence Immunoassay
ERα	Estrogen Receptor alpha
FMD	Flow-Mediated Dilation
