# Supplementary material for: Metabolic and Endocrine Alterations in Underweight and Normal-Weight Women with Functional Hypothalamic Amenorrhea
Source: J Clin Med. 2025 Oct 7;14(19):7082. doi: 10.3390/jcm14197082 (PMC12524805; doi:10.3390/jcm14197082)
Supplement: Supplementary file 1 [file jcm-14-07082-s001.zip › Figure S2.pdf]

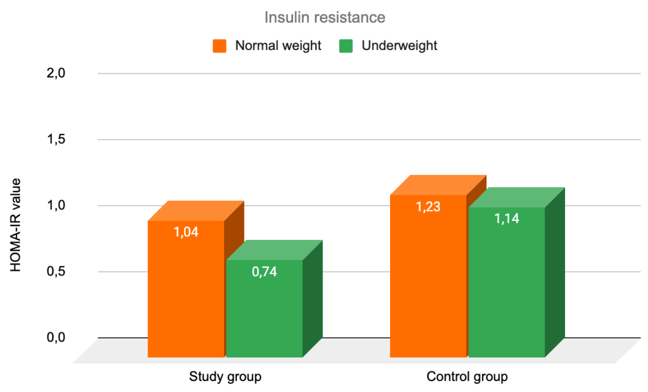

(a) Insulin resistance

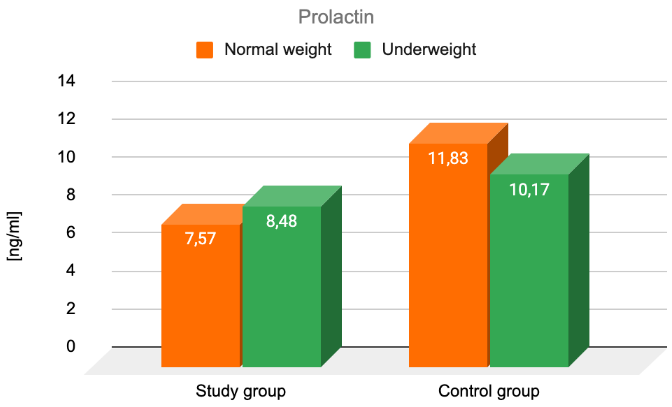

(b) Prolactin

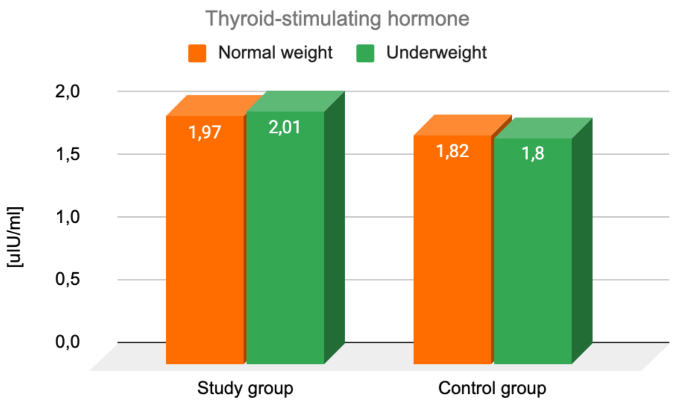

(c) Thyroid-stimulating hormone

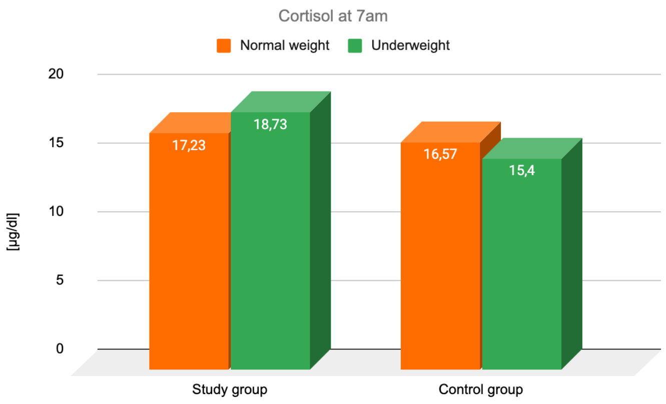

(d) Cortisol at 7am

Figure S2. Results of **(a)** insulin resistance, **(b)** prolactin, **(c)** TSH and **(d)** cortisol in the subgroups.
