# Supplementary material for: Metabolic and Endocrine Alterations in Underweight and Normal-Weight Women with Functional Hypothalamic Amenorrhea
Source: J Clin Med. 2025 Oct 7;14(19):7082. doi: 10.3390/jcm14197082 (PMC12524805; doi:10.3390/jcm14197082)
Supplement: Supplementary file 1 [file jcm-14-07082-s001.zip › Figure S1.pdf]

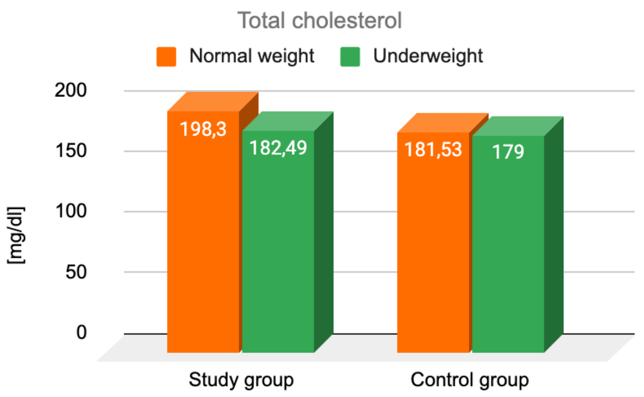

(a) Total cholesterol

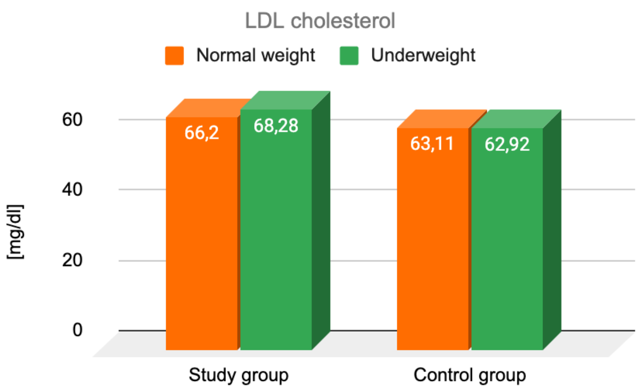

(b) LDL cholesterol

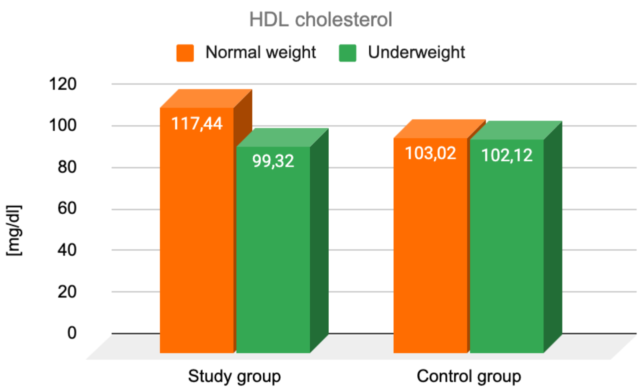

(c) HDL cholesterol

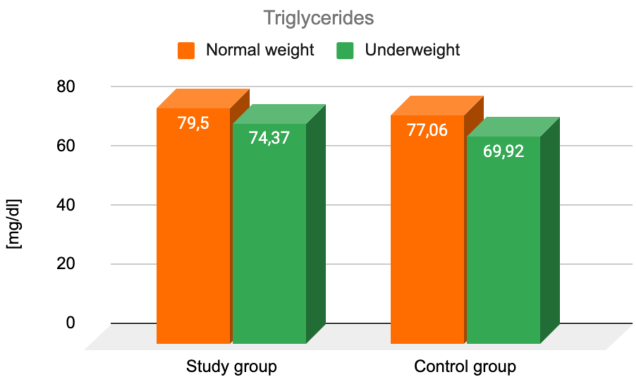

(d) Triglycerides

Figure S1. Results of **(a)** total, **(b)** LDL, **(c)** HDL cholesterol and **(d)** triglycerides in the subgroups.
